# Quantifying the effects of ice hockey upper body pads on mobility and comfort

**DOI:** 10.1016/j.isci.2023.108606

**Published:** 2023-11-30

**Authors:** Yiwei Wu, Yanfei Shen, Yinsheng Tian, Qi Chen, Lixin Sun

**Affiliations:** 1 AI Sports Engineering Lab, School of Sports Engineering, Beijing Sport University, Beijing 100084, China; 2 Sports Engineering Research Center, China Institute of Sport Science, Beijing 100061, China

**Keywords:** Sports medicine, Physics, Mechanics

## Abstract

Ice hockey is a high-intensity sport in which pads such as shoulder and elbow pads (S/EPs) are necessary to help players avoid injury. However, they can also affect mobility and comfort, thereby affecting players’ on-ice performance. We aimed to quantify the effects of S/EPs on mobility and comfort by comparing the range of motion (ROM) of nine elite college-level ice hockey players performing static (nine single-DOF upper-body movements) and dynamic (wrist and slap shots) tasks under six pad conditions (no S/EPs and five types of S/EPs). We also analyzed the relationship between ROM and subjective comfort to provide an objective comfort evaluation of hockey pads. Five types of S/EPs restrict ROM at different levels, imposing additional mobility restrictions. We found significant differences among the five types and a high correlation between comfort and ROM. We conducted a comprehensive evaluation of the impact of ice hockey pads on mobility and comfort.

## Highlights

• Comprehensive evaluation revealed ice hockey pad’s impact on mobility and comfort
• Five S/EP types limit ROM at various levels, further restricting mobility
• We found significant differences among the five types of S/EPs
• High correlation was identified between comfort and ROM

## Graphical abstract

**Figure:**
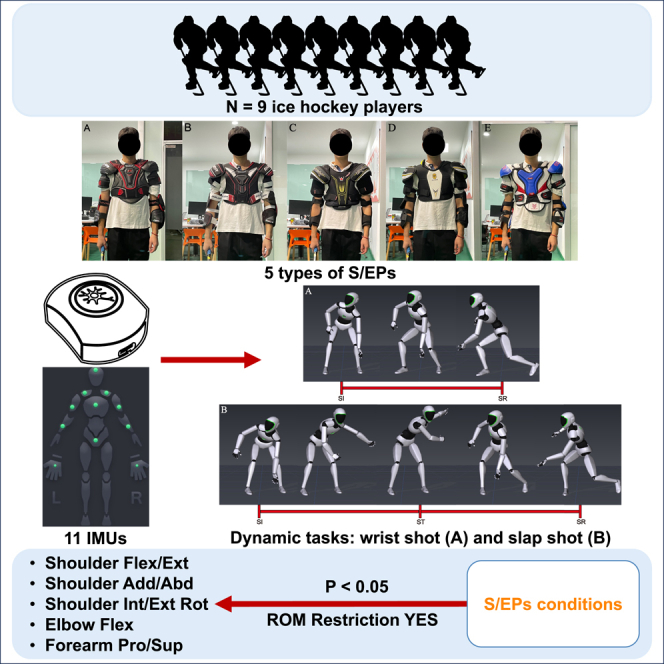

## Introduction

Ice hockey is a high-intensity sport that is associated with a high risk of injury.^1^ These injuries can be caused by unintended collisions, high velocities, rapid changes in direction, or contact with boards, sticks, or pucks.^2^^,^^3^ A study of ice hockey injuries among players in the US National Hockey League, Canadian, and European leagues showed that the knee, shoulder, groin, and back were the main areas of injury, accounting for 40%, 20%, 15%, and 10%, respectively.^4^ Therefore, players must wear personal protective equipment (PPE) all over their bodies to avoid severe injuries.^5^ According to the IIHF (International Ice Hockey Federation), PPE used in ice hockey includes helmets, neck guards, shoulder pads, elbow pads, gloves, pants, and shin guards.^6^^,^^7^ Initially, ice hockey pads were mainly made of sponge, plastic, and leather, which made them bulky. With technological advances, the materials used in these pads range from those made of ordinary foam and hard plastic to those with advanced performance foam (e.g., expanded polypropylene) to absorb the game’s blunt force and high-speed impacts and achieve light weight.

Several studies have shown that PPE is inconvenient to use, limits mobility,^8^^,^^9^ and makes people feel uncomfortable owing to thermal stress.^10^^,^^11^^,^^12^^,^^13^ Ice hockey pads are a type of PPE that may also hamper normal activities owing to differences in material, size, and suitability for the wearer. As the primary purpose of these pads is to better protect players, they may not have been designed with much regard to how they would affect mobility and comfort. Proper sports equipment development should reflect factors that affect performance and injury.^14^ Among the various PPE used in ice hockey, helmets have been relatively well studied,^15^^,^^16^^,^^17^ whereas few studies have been performed on how ice hockey pads affect mobility and comfort. In the case of ice hockey pads, restricting the range of motion (ROM) can ultimately affect a player’s comfort. Krause et al.^18^ found that ice hockey neck laceration protectors (NLPs) can reduce the risk of neck laceration but appear to affect cervical ROM. Frayne et al.^19^ quantified the ROM of four ice hockey goalie leg pads during butterfly maneuvers. The results indicated that the stiff, wide leg pads caused much more external hip rotation than the flex-tight or flex-wide ones. Studies on NLPs and goalie leg pads have shown that ice hockey pads can affect mobility.

However, no study has investigated ice hockey upper-body pads (i.e., shoulder and elbow pads [S/EPs]) designed to protect the trunk, shoulder, and elbow from injuries.^20^ Although these hockey pads are continually being improved, most innovations come from feedback from professional players and the equipment regulations of the IIHF. While international standards (ISO 10256, protective equipment for use in ice hockey) exist for evaluating ice hockey pads, they are mostly related to their size, protective area, resistance to impact, and cutting.^21^ However, research on the physical effects of ice hockey upper-body pads on players is lacking. Numerous ice hockey players believe that such pads can seriously compromise their performance.^22^ Considering that players must perform at a high level in fast-paced competitions, high mobility and comfort are necessary. Previous studies have predominantly utilized subjective methods to assess the effects of PPE on mobility and comfort.^23^^,^^24^^,^^25^^,^^26^ However, this approach restricts the evaluation cycle, impeding product design and standardization. Research on PPE has emphasized the importance of investigating how PPE affects the capacity to execute fundamental movements.^27^ Such analyses are vital in developing PPE that balances comfort, effectiveness, and protection while upholding performance.^28^ Typically, the ergonomic performance evaluation of PPE involves static and dynamic assessments,^29^ and analyzing ROM is a common practice to measure changes in mobility and movement patterns.^30^

**Figure 1:**
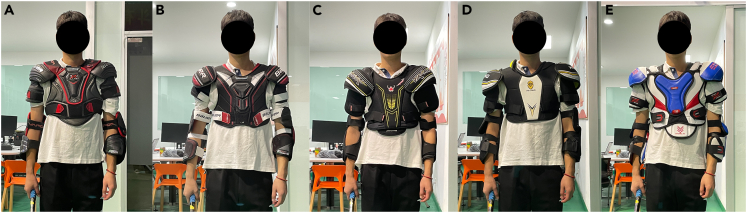
Five types of assessed S/EPs (A) Bauer Vapor (1X LITE, Bauer Hockey Inc., USA). (B) Bauer NSX (S18, Bauer Hockey Inc., USA). (C) Vix-Max (Premium, Vik-Max Sports Equipment Inc., China). (D) Heilong (Premium, Heilong International Ice & Snow Equipment Inc., China). (E) IBX (X730, Icebreaker Sports Equipment Inc., China) S/EPs, shoulder and elbow pads.

**Figure 2:**
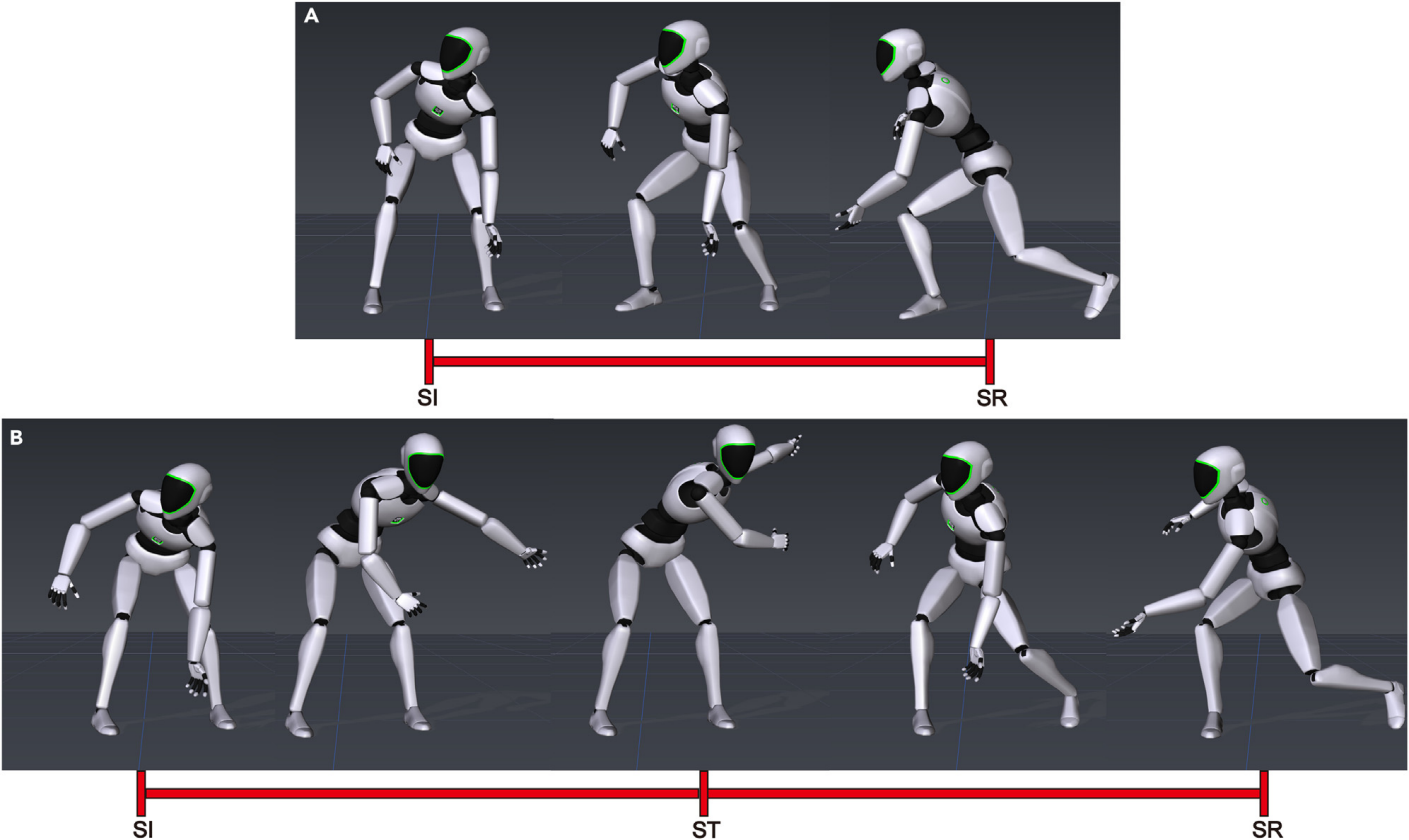
Three-dimensional (3D) diagrammatic images of a participant’s entire body motion during the wrist shot (A) and slap shot (B). Two shooting events were identified in the wrist shot (i.e., shot initiation (SI) and shot release (SR)^31^) and three in the slap shot (i.e., shot initiation (SI), swing top (ST), and shot release (SR)^32^).

We quantified the effect of hockey pads (five types of S/EPs, Figure 1) on mobility by comparing the ROM collected from nine elite college-level hockey players completing static (nine single-DOF upper-body movements) and dynamic (wrist and slap shots,^31^^,^^32^; Figure 2) tasks under six pad conditions (no S/EPs as the control condition and five S/EPs). In this study, we expanded the static measurement of the ROM to include nine single-DOF motions to provide a comprehensive analysis of the impact of S/EPs on mobility. We chose wrist and slap shots as dynamic tasks to quantify the effect of hockey pads on player mobility in a competitive game more realistically. Furthermore, we examined players’ perceptions of the comfort of ice hockey pads using a 5-point Likert^33^ type scale. To quantify the effect of hockey pads on comfort, we analyzed the relationship between the ROM and subjective comfort. When players were wearing hockey pads, we observed significant limitations in the ROM of specific movements, including shoulder flexion, shoulder abduction, and elbow flexion. These findings can guide the design and improvement of hockey pads. In addition, we found an extremely high correlation between the comprehensive upper-body ROM and comfort evaluation results, indicating that this method can objectively and accurately assess the comfort of ice hockey pads.

## Results

The upper-body ROM data of nine ice hockey players performing 11 tasks (nine static and two dynamic tasks) under six different S/EP conditions resulted in 594 ROM measurements for analysis (9 participants × 11 tasks × 6 conditions). The Shapiro–Wilk test confirmed that the upper-body ROM data were normally distributed (p < 0.05) across all investigated tasks under the six different S/EP conditions.

**Figure 3:**
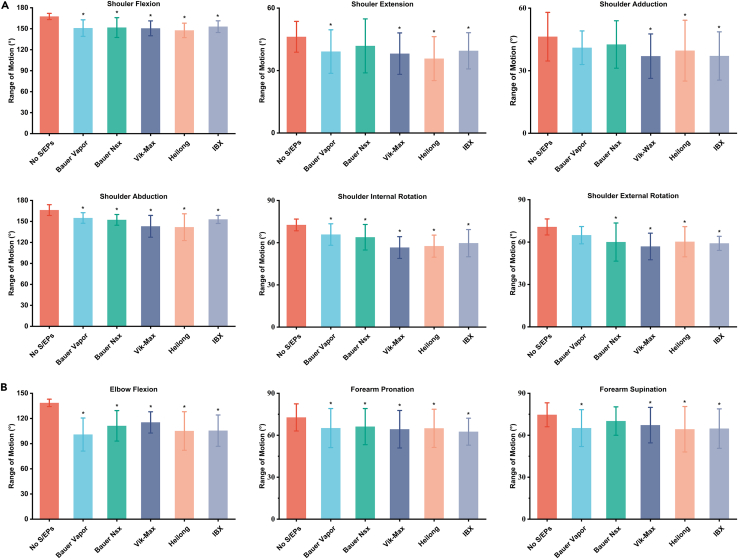
Mean shoulder (A) and elbow (B) ROM (M±SD) for each S/EPs condition during all investigated static tasks The asterisk represents a significant difference (p < 0.05) compared with the no-shoulder/elbow pad condition (no S/EPs). Note that from left to right are No S/EPs, Bauer Vapor, Bauer Nsx, Vik-Max, Heilong, and IBX. ROM, range of motion; M ± SD, mean ± standard deviation; S/EPs; Shoulder and elbow pads.

**Figure 4:**
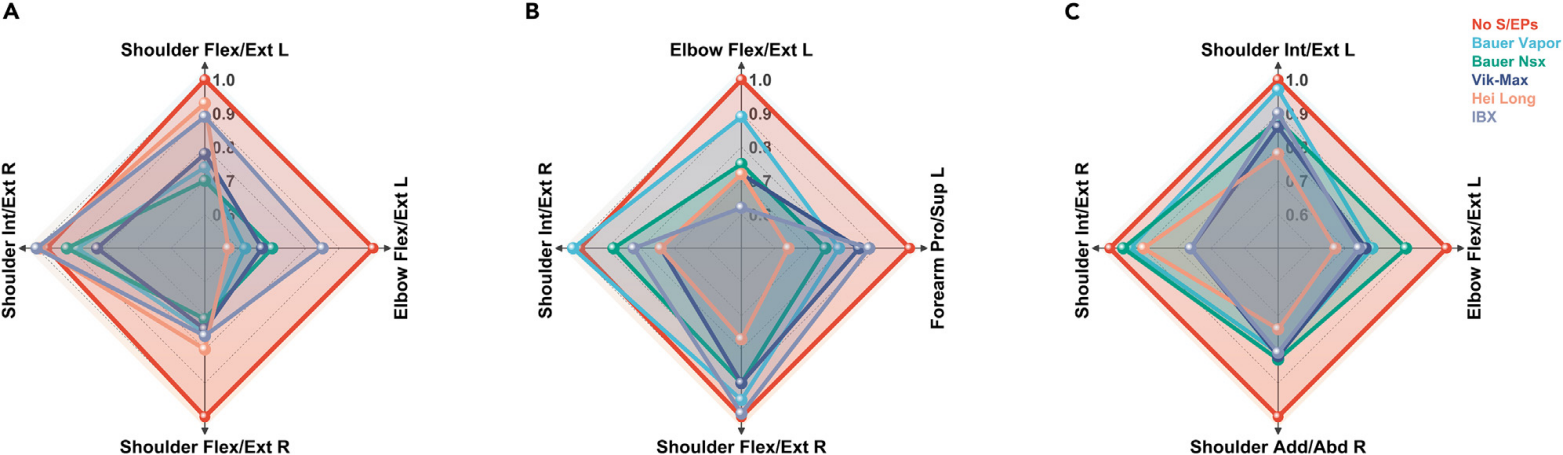
Movement pattern in different S/EP conditions for dynamic tasks The ROM in the no S/EPs condition was used as the criterion for comparison. (A–C) ROM was measured from SI to SR in the wrist shot of (A), from SI to ST in the slap shot of (B), and from ST to SR in the slap shot of (C). S/EPs; shoulder and elbow pads; ROM, range of motion; SI, shot initiation; SR, shot release; ST, swing top.

**Figure 5:**
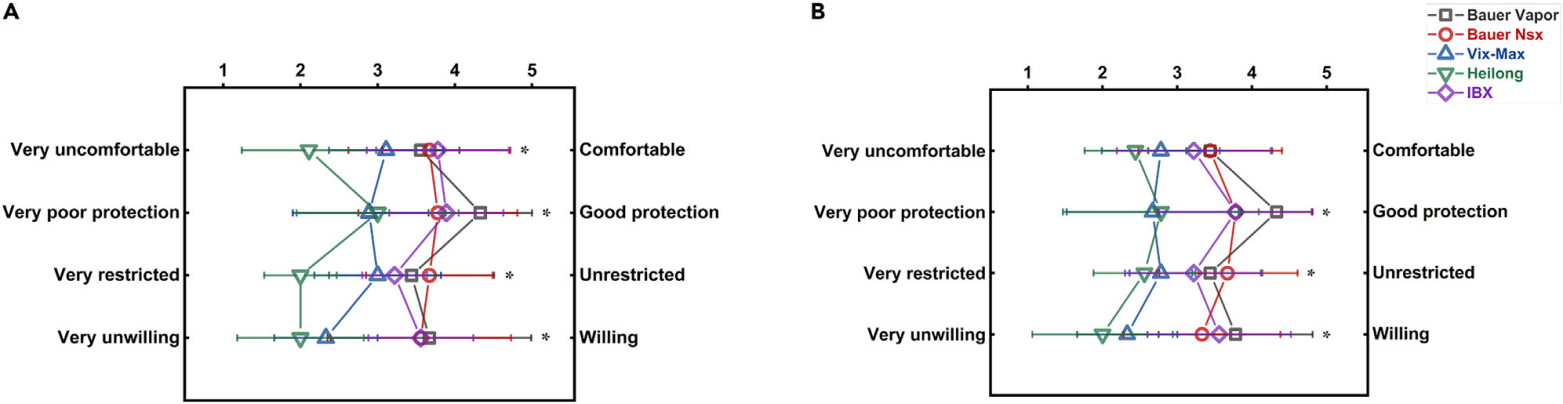
Subjective evaluation results (A and B) The evaluation results represent nine responses to four subjective questions posed for each S/EPs as follows: (Q1) This shoulder or elbow pad is comfortable; (Q2) This shoulder or elbow pad is safe; (Q3) This shoulder or elbow pad limits my shoulder or elbow motion; (Q4) I would wear this specific shoulder or elbow pad if wearing one is optional. The asterisk represents comparisons between five types of S/EPs that were statistically significant (p < 0.05). S/EPs; shoulder and elbow pads.

**Figure 6:**
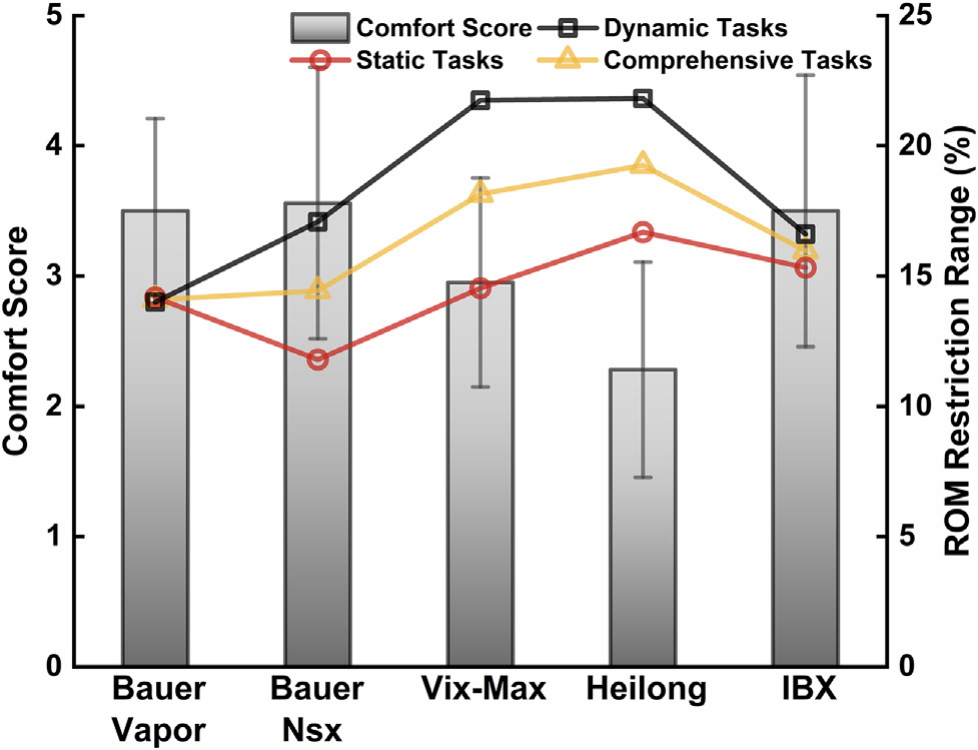
Results of comfort and upper-body (i.e., shoulder and elbow) ROM restrictions in five types of S/EPs ROM, range of motion; S/EPs; shoulder and elbow pads.

Figure 3 shows the mean shoulder and elbow ROM for each S/EPs condition during all the investigated static tasks. The descriptive statistics (MSD), one-way repeated measures analysis of variance (ANOVA), and post-hoc analysis results for shoulder and elbow ROM during different S/EP conditions, with the effect size for ANOVA, are reported in Tables 1 and S1. Figure 4 shows the normalized ROM with significant differences, as indicated by a one-way repeated measures ANOVA in all examined dynamic tasks. The results of the one-way repeated measures ANOVA, post-hoc analysis, and descriptive statistics for elbow and shoulder ROM under various S/EP conditions during all dynamic tasks are shown in Tables 2, 3, and S2–S4. Figure 5 shows the participants’ subjective survey results, and subjective evaluation scores of different types of S/EPS during static and dynamic tasks are presented in Table S5. Figure 6 show the results of the subjective comfort evaluation and the objective ROM restriction range.

**Table 1:** Comparison of independent variables for different types of S/EPs during all investigated static tasks (i.e., descriptive statistics and one-way repeated measures ANOVA outcomes of shoulder and elbow ROM in different S/EP conditions)

Variables		No S/EPs	Bauer Vapor	Bauer Nsx	Vik-Max	Heilong	IBX	p	F	$\eta_{p}^{2}$
Shoulder	Flex	167.6 ± 4.5^2,3,4,5,6^	150.9 ± 11.8^1^	151.7 ± 14.2^1^	150.6 ± 10.6^1^	147.6 ± 10.3^1^	153.0 ± 8.3^1^	<0.01	7.362	0.479
	Ext	46.2 ± 7.4^2,4,5,6^	39.1 ± 10.5^1^	41.8 ± 13.0^5^	38.1 ± 9.9^1^	35.7 ± 10.6^1,3^	39.5 ± 8.7^1^	0.013	3.345	0.295
	Add	45.7 ± 12.2^4,5,6^	41.1 ± 8.5	41.7 ± 11.7	36.3 ± 11.0^1^	38.3 ± 15.2^1^	36.4 ± 12.0^1^	0.016	3.152	0.259
	Abd	166.2 ± 7.7^2,3,4,5,6^	154.9 ± 7.6^1,4,5^	152.2 ± 7.7^1,5^	143.0 ± 15.6^1,2^	141.8 ± 19.2^1,2,3,6^	152.9 ± 5.8^1,5^	<0.01	6.653	0.454
	Int Rot	70.7 ± 5.7^3,4,5,6^	65.8 ± 7.6^1,4,5,6^	63.9 ± 9.1^1,4,5^	56.5 ± 7.7^1,2,3^	57.5 ± 7.8^1,2,3^	59.7 ± 9.7^1,2^	<0.01	9.466	0.542
	Ext Rot	138.6 ± 4.4^2,3,4,5,6^	64.9 ± 6.1^4^	60.0 ± 13.5^1^	56.9 ± 9.4^1,2^	60.3 ± 10.7^1^	59.1 ± 5.0^1^	<0.01	4.378	0.354
Elbow	Flex	77.7 ± 9.7^2,3,4,5,6^	100.9 ± 19.7^1,4^	111.2 ± 18.1^1^	115.3 ± 12.7^1,2^	105.1 ± 23.0^1^	105.5 ± 18.7^1^	<0.01	10.622	0.570
	Pro	77.7 ± 9.7^2,3,4,5,6^	65.1 ± 14.0^1^	66.2 ± 12.9^1^	64.3 ± 13.4^1^	65.0 ± 13.7^1^	62.6 ± 9.6^1^	0.035	3.651	0.313
	Sup	74.6 ± 8.6^2,4,5,6^	65.1 ± 13.2^1^	70.1 ± 10.2	67.2 ± 12.7^1^	64.3 ± 16.2^1^	64.8 ± 14.1^1^	0.017	3.175	0.284

Note that 1, 2, 3, 4, 5, and 6 are significantly different from No S/EPs, Bauer Vapor, Bauer NSX, Vik-Max, Heilong, and IBX, respectively. S/EPs; shoulder and elbow pads; ROM, range of motion; ANOVA, analysis of variance.

**Table 2:** Comparison of independent variables for different types of S/EPs measured from SI to SR during the wrist shot (i.e., descriptive statistics and one-way repeated measures ANOVA outcomes of shoulder and elbow ROM in different S/EPs conditions)

Variables		No S/EPs	Bauer Vapor	Bauer Nsx	Vik-Max	Heilong	IBX	p	F	$\eta_{p}^{2}$
Shoulder	Flex/Ext L	26.7 ± 8.5^2,3,4^	19.7 ± 4.2^1,4^	18.7 ± 5.9^1,5,6^	20.8 ± 7.6^1^	24.8 ± 7.4^2,3^	23.7 ± 8.3^3^	0.011	3.473	0.303
	Add/Abd L	15.6 ± 7.0	14.8 ± 4.8	14.4 ± 6.7	13.2 ± 5.1	14.4 ± 6.0	14.1 ± 6.5	0.847	0.398	0.047
	Int/Ext Rot L	33.9 ± 16.4	32.2 ± 15.2	31.8 ± 19.1	31.1 ± 17.7	33.7 ± 16.4	30.3 ± 16.2	0.484	0.910	0.102
	Flex/Ext R	38.1 ± 11.7^2,3,4,5,6^	28.7 ± 8.0^1^	26.9 ± 9.1^1^	27.6 ± 6.0^1^	30.5 ± 8.8^1^	29.0 ± 8.0^1^	0.048	2.475	0.236
	Add/Abd R	25.6 ± 12.0	24.8 ± 11.1	21.9 ± 8.4	25.7 ± 9.6	23.7 ± 9.8	25.3 ± 10.8	0.334	1.177	0.144
	Int/Ext Rot R	29.5 ± 19.2^4^	26.7 ± 19.1^6^	27.5 ± 17.5	24.8 ± 11.9^1,5,6^	29.9 ± 18.7^4^	30.3 ± 15.3^2,4^	0.037	2.655	0.228
Elbow	Flex L	29.4 ± 13.3^2,3,4,5^	18.3 ± 7.1^1,6^	20.6 ± 6.2^1^	19.6 ± 13.2^1^	16.8 ± 8.1^1,6^	25.1 ± 12.1^2,5^	0.020	4.500	0.360
	Pro/Sup L	16.5 ± 13.8	14.3 ± 8.5	16.0 ± 8.9	14.9 ± 8.8	13.0 ± 6.8	13.6 ± 7.3	0.363	1.125	0.123
	Flex R	14.3 ± 4.8	13.4 ± 4.5	12.8 ± 2.2	12.4 ± 3.3	14.7 ± 3.2	14.7 ± 4.2	0.306	1.275	0.137
	Pro/Sup R	10.8 ± 4.5	9.9 ± 4.0	10.1 ± 5.7	11.5 ± 4.6	10.5 ± 4.4	9.8 ± 4.8	0.867	0.279	0.034

Note that 1, 2, 3, 4, 5, and 6 are significantly different from No S/EPs, Bauer Vapor, Bauer NSX, Vik-Max, Heilong, and IBX, respectively. S/EPs; shoulder and elbow pads; ROM, range of motion; ANOVA, analysis of variance; Flex/Ext L, flexion/extension left; Add/Abd L, adduction/abduction left; Int/Ext Rot L, internal/external rotation left; Flex/Ext R, flexion/extension right; Add/Abd R, adduction/abduction right; Int/Ext Rot R, internal/external rotation right; Flex L, flexion left; Pro/Sup L, pronation/supination left; Flex R, flexion right; Pro/Sup R, pronation/supination right.

**Table 3:** Comparison of independent variables for different types of S/EPs during the slap shot (i.e., descriptive statistics and one-way ANOVA repeated measures outcomes of shoulder and elbow ROM in different S/EP conditions)

Variables			No S/EPs	Bauer Vapor	Bauer Nsx	Vik-Max	Heilong	IBX	p	F	$\eta_{p}^{2}$
SI-ST	Shoulder	Flex/Ext L	41.5 ± 24.0	38.1 ± 26.8	35.3 ± 21.8	38.0 ± 22.2	35.5 ± 25.0	41.2 ± 19.5	0.354	1.143	0.125
		Add/Abd L	23.7 ± 7.7	23.2 ± 7.6	22.7 ± 5.7	19.9 ± 5.3	23.3 ± 7.0	20.8 ± 60.9	0.129	1.830	0.186
		Int/Ext Rot L	59.7 ± 21.6	60.2 ± 17.9	51.5 ± 11.4	58.2 ± 23.8	50.8 ± 17.9	56.5 ± 20.9	0.453	0.885	0.100
		Flex/Ext R	23.5 ± 8.5^5^	22.4 ± 10.4^5^	21.2 ± 5.3	21.0 ± 8.3	18.0 ± 6.9^1,2,6^	23.1 ± 8.7^5^	0.037	2.642	0.248
		Add/Abd R	25.0 ± 6.4	23.8 ± 6.4	25.3 ± 6.7	23.6 ± 9.2	22.3 ± 5.0	21.9 ± 5.1	0.352	1.148	0.125
		Int/Ext Rot R	35.4 ± 10.5^4,5^	36.0 ± 10.1^4,5,6^	31.8 ± 9.2	26.5 ± 8.8^1,2^	26.6 ± 7.2^1,2^	29.4 ± 11.7^2^	0.014	3.291	0.291
	Elbow	Flex/Ext L	28.6 ± 14.4^3,4,5,6^	25.4 ± 13.3^6^	21.6 ± 15.5^1^	20.7 ± 15.1^1^	20.6 ± 13.6^1^	17.7 ± 14.1^1,2^	<0.01	4.723	0.371
		Pro/Sup L	18.9 ± 8.2^2,3,4^	14.9 ± 6.5^1^	14.1 ± 6.1^1^	16.0 ± 5.5^1,5^	12.2 ± 3.0^4,6^	16.6 ± 6.0^5^	0.021	3.011	0.273
		Flex/Ext R	34.6 ± 15.8	30.8 ± 14.2	34.0 ± 18.3	34.9 ± 19.5	33.3 ± 16.9	34.3 ± 17.1	0.545	0.690	0.079
		Pro/Sup R	20.3 ± 9.7	18.4 ± 7.4	20.1 ± 6.9	21.1 ± 11.1	20.2 ± 10.8	19.4 ± 5.9	0.835	0.193	0.024
ST-SR	Shoulder	Flex/Ext L	47.3 ± 21.4	38.43 ± 20.6	43.7 ± 18.8	43.7 ± 18.9	43.1 ± 21.5	45.0 ± 21.7	0.151	2.174	0.214
		Add/Abd L	26.4 ± 9.4	26.1 ± 9.2	24.1 ± 8.4	26.4 ± 11.1	25.2 ± 10.7	24.7 ± 8.4	0.851	0.393	0.047
		Int/Ext Rot L	52.6 ± 17.8^5^	51 ± 13.1^5^	46.4 ± 6.82	45.3 ± 14.1	41.1 ± 9.2^1,2^	47.3 ± 16.2	0.050	2.445	0.234
		Flex/Ext R	28.6 ± 14.1	29.0 ± 14.1	26.8 ± 12.7	28.6 ± 10.2	23.6 ± 8.5	25.4 ± 14.0	0.465	0.944	0.119
		Add/Abd R	29.2 ± 8.9^2,3,4,5,6^	23.6 ± 5.1^1^	24.2 ± 3.1^1^	23.8 ± 5.8^1^	21.7 ± 5.7^1^	23.8 ± 5.7^1^	0.043	2.543	0.241
		Int/Ext Rot R	51.5 ± 21.1^4,6^	48.3 ± 21.5^4,6^	49.5 ± 21.3^4,6^	39.0 ± 21.6^1,2,3^	46.5 ± 16.9	39.1 ± 20.6^1,2,3^	0.018	3.175	0.312
	Elbow	Flex/Ext L	26.1 ± 10.6^2,4,5,6^	20.4 ± 7.1^1^	23.0 ± 10.7^5^	19.7 ± 14.4^1^	17.4 ± 10.6^1,3^	19.3 ± 8.5^1^	<0.01	3.576	0.309
		Pro/Sup L	17.9 ± 7.7	16.0 ± 7.4	14.8 ± 5.7	18.7 ± 10.0	14.6 ± 5.9	18.1 ± 10.5	0.355	1.129	0.158
		Flex/Ext R	43.4 ± 13.6	37.2 ± 18.9	43.3 ± 22.3	41.9 ± 20.2	42.9 ± 22.5	40.2 ± 18.9	0.422	0.922	0.103
		Pro/Sup R	24.4 ± 12.8	19.3 ± 7.8	24.1 ± 4.9	22.3 ± 6.3	24.1 ± 6.6	21.8 ± 7.5	0.571	0.778	0.089

Note that 1, 2, 3, 4, 5, and 6 are significantly different from No S/EPs, Bauer Vapor, Bauer NSX, Vik-Max, Heilong, and IBX, respectively. S/EPs; Shoulder and elbow pads; ROM, range of motion; ANOVA, analysis of variance; SI, shot initiation; SR, shot release; ST, swing top; Flex/Ext L, flexion/extension left; Add/Abd L, adduction/abduction left; Int/Ext Rot L, internal/external rotation left; Flex/Ext R, flexion/extension right; Add/Abd R, adduction/abduction right; Int/Ext Rot R, internal/external rotation right; Flex L, flexion left; Pro/Sup L, pronation/supination left; Flex R, flexion right; Pro/Sup R, pronation/supination right.

## Discussion

### Static ROM analysis

A one-way repeated measure ANOVA revealed significant differences between the five types of S/EP and the control condition (p < 0.05, mean $\eta_{p}^{2}$ = 0.394) during all investigated static tasks, except for the Bauer NSX for shoulder extension (p = 0.124), Bauer Vapor and Bauer NSX for shoulder adduction (p = 0.068 and 0.194, respectively), Bauer Vapor for shoulder external rotation (p = 0.093), and Bauer Nsx for forearm supination (p = 0.169). Post-hoc analyses indicated no significant difference (p > 0.05) in the ROM of shoulder flexion, shoulder adduction, forearm pronation, and forearm supination among the five types of S/EP. However, the restrictions were more pronounced for movements with a greater range of motion, specifically shoulder flexion, shoulder abduction, and elbow flexion.

Shoulder movements were more limited in the transverse plane than in the other planes of motion by the shoulder pads. Internal rotation of the shoulder was limited from 9.4% (Bauer Vapor) to 22.1% (Vik-Max), whereas external rotation was limited from 8.2% (Bauer Vapor) to 19.5% (Vik-Max). The Vix-Max allowed for less rotational movement when compared to the other four shoulder pads and was found to be the most restrictive S/EPs, limiting shoulder ROM by 17.3% on average, compared to the Heilong (16.6%), IBX (14.3%), Bauer NSX (10.6%), and Bauer Vapor (10.0%), when considering the total ROM value. The Vix-Max had the most restrictive shoulder pads, whereas the IBX had the most restrictive elbow pads. On average, the overall shoulder motion was limited by 17.3% with Vix-Max and elbow motion by 17.0% with IBX. In contrast, the Bauer Vapor (with an average restriction of 10%) and Bauer NSX (with an average limitation of 11.6%) were the least restrictive shoulder and elbow pads, respectively. Shoulder abduction was the most restricted shoulder movement, with a mean value of 17.3° less than in the no-S/EP condition, followed by shoulder flexion, which was 16.8° less. This may be due to the shoulder caps above the shoulder pads, which restrict upward movement of the shoulder. Although “high sticking” (players carrying sticks above the normal height of their opponents’ shoulders)^34^ is not allowed in ice hockey, the restricted shoulder movement found in static tasks is still a non-negligible concern.

Elbow movements were more limited in the sagittal plane than in the other planes of motion by the elbow pads. Elbow flexion was limited by 16.8–27.2% with different elbow pads compared with no S/EPs. With different elbow pads, arm pronation and supination were limited by 6–14%. Bauer Vapor significantly limited elbow flexion compared with the other four S/EPs, particularly in the sagittal plane. Based on the total ROM value, IBX was the most restrictive, limiting elbow ROM by 17% on average, compared to other S/EPs such as the Bauer Vapor (16.8%), Heilong (16.2%), Vix-Max (12.8%), and Bauer NSX (11.6%). The effect size indicated that elbow flexion was significantly influenced by the type of pad (average $\eta_{p}^{2}$ = 0.570), resulting in a mean reduction of 22.4°, which may have restricted the bicep guard and elbow cap of the elbow pad. Forearm protonation and supination were less affected by S/EPs type (average $\eta_{p}^{2}$ = 0.299), with a mean reduction of 11.1°. Bauer Vapor caused the least shoulder movement restriction but more elbow limitation (second only to IBX) than the other S/EPs. This may be due to the higher level of protection provided by the Bauer Vapor, which covers a larger arm area, resulting in a greater restriction of elbow movement. Meanwhile, players rated the Bauer Vapor as the most protective, indicating that the added protection from the hockey pads restricted the static ROM in the upper body.

The evaluation of static tasks aims to investigate the influence of PPE on the mobility of a single joint,^35^ the movements of which were found to be limited by the S/EPs. The static task test in this study was beneficial for obtaining information about design features in specific parts of the S/EPs and ergonomic performance. We conclude that the current commercially available ice hockey PPE improves protection by increasing the protective area. However, this limits the mobility of players. These limitations in mobility may result in players exerting greater physical effort, leading to increased metabolic rate, body temperature, and perspiration, which can contribute to physical strain.^9^

### Dynamic ROM analysis

One-way repeated measures ANOVA was conducted to assess the impact of variations in S/EPs on the ROM of different joint movements during wrist and slap shots. The results indicated a significant effect on the ROM of left shoulder flexion/extension, left elbow flexion/extension, right shoulder flexion/extension, and right shoulder internal/external rotation during the wrist shot (p = 0.011–0.048, $\eta_{p}^{2}$ = 0.228–0.360), left elbow flexion/extension, left forearm pronation/supination, right shoulder flexion/extension, and right shoulder internal/external rotation during the SI (shot initiation) to ST (swing top) in slap shot (p = 0.002–0.037, $\eta_{p}^{2}$ = 0.248–0.371), and left shoulder internal/external rotation, left elbow flexion/extension, right shoulder adduction/abduction, and right shoulder internal/external rotation during the ST to SR (shot release) in slap shot (p = 0.018–0.050, $\eta_{p}^{2}$ = 0.234–0.312) (Figure 6). The results of the post-hoc analysis showed that there were significant differences (p < 0.05) in most motion planes between the five S/EP conditions, that is, left and right shoulder flexion/extension, left and right shoulder internal/external rotation, left elbow flexion, and left forearm protonation/supination, during all shooting trials.

In the wrist shot, sagittal plane movements were more limited by S/EPs than the other motion planes, with left shoulder flexion/extension limitation compared to no S/EPs ranging from Heilong (7.1%) to Bauer NSX (30.2%), right shoulder flexion/extension ranging from Heilong (19.8%) to Bauer NSX (29.3%), and left elbow flexion ranging from IBX (14.7%) to Heilong (42.9%). The S/EPs condition had a more significant limitation of the left elbow flexion/extension ROM than the other joint motions, with a mean limitation of 31.7%. Highlighting sagittal plane movements, the Bauer NSX significantly restricted shoulder flexion/extension motion, and Heilong significantly restricted left elbow flexion/extension motion more than the other four S/EPs. Considering the total ROM value, the Vix-Max was the most restrictive, with an average ROM limitation of 24.6%.

During the slap shot, frontal plane movements were more limited by S/EPs than the other motion planes, with right shoulder adduction/abduction limitations compared to no S/EPs, ranging from Bauer NSX (17.2%) to Heilong (25.8%). Heilong significantly restricted the right shoulder adduction/abduction motion compared with the other four SPs. Considering the total ROM value, Heilong had the most restrictive type of S/EPs, with an average ROM limitation of 24.0% measured from an entire slap shot (i.e., SI to ST and ST to SR), compared with Vix-Max (20.3%), IBX (18.6%), Bauer NSX (13.5%), and Bauer Vapor (8.8%).

During dynamic tasks, significant ROM limitations were found in shoulder flexion/extension, shoulder internal/external rotation, right shoulder adduction/abduction, left elbow flexion/extension, and left forearm pronation/supination. The magnitude of the restrictions varied. A study of ice hockey wrist shot accuracy by Michaud-Paquette et al.^31^ found that more accurate shooters produced greater ROM in forearm pronation/supination (approximately 25°). Similarly, we found a significant limitation in forearm motion by S/EPs during shooting trials. A concern is that limited forearm motion may negatively affect shooting accuracy. Blackledge et al.^36^ found that PPE affects movement posture, which may further affect performance. In addition, restricted mobility may lead players to apply greater force and cause more physical strain^9^ during competitions. Moreover, higher perspiration rates from restricted mobility resulted in greater moisture accumulation within ice hockey pads. This, in turn, directly enhances the friction between the skin and the protective liner, further exacerbating the garment’s binding and restriction of movement, leading to additional perspiration and rapid physical exhaustion.^37^

Bauer Vapor and IBX were the least restrictive, with average restrictions of 12% and 22.8%, respectively. The effect size revealed that the elbow pad type largely affected the left elbow flexion (average $\eta_{p}^{2}$ = 0.347), with a mean reduction of 27.1°. Considering the total ROM, Heilong was the most restrictive, with an average ROM limitation of 22.9%. We speculate that the weight of the S/EPs could affect mobility. Heilong is the heaviest of the five types of S/EPs, weighing 1.57 kg, followed by IBX at 1.51 kg, Vix-Max at 1.24 kg, Bauer NSX at 1.19 kg, and Bauer Vapor at 1.15 kg, which indicates that the added weight of the S/EPs restricted ROM. Similarly, a review by Tochihara et al.^29^ also reported that ROM decreased as the weight of PPE increased. Our results are similar to those of other investigations of PPE limitations on mobility and extend previous findings on ice hockey protectors. A report^5^ indicated that ice hockey players expressed many complaints about the shape and construction of PPE. However, they were satisfied with the overall safety of the PPE. Players experience significant discomfort from the pads sliding down or moving around. To date, most S/EPs are secured using elastic straps and Velcro.^7^ Therefore, we speculate that sliding protector during movement may limit mobility.

As discussed above, the use of S/EPs reduced the mobility of the players performing both shooting trials. We found that the S/EP conditions significantly negatively impacted the upper-body ROM, particularly in the sagittal and frontal plane joint movements. Dynamic tasks have different outcomes from static tasks because they involve concurrent movements at multiple joints. However, dynamic tasks provide a more realistic depiction of the effect of ice hockey pads on players during competitive games.

### Comprehensive comfort evaluation

According to the subjective survey results of all the participants (Table S5) and post-hoc Wilcoxon signed-rank tests for the five types of S/EPs (Figure 5), most differences among the five types of S/EPs were statistically significant (p < 0.05), except for the question “This elbow pad is comfortable.” Based on the results of the study, the players ranked the IBX as the most comfortable shoulder pad, with an average score of 3.8, followed by the Bauer NSX (3.7) and the Bauer Vapor (3.6). Similarly, for elbow pads, the players ranked Bauer Vapor and Bauer NSX as the most comfortable, with an average score of 3.4, followed by IBX (3.2). Regarding safety, Bauer Vapor was ranked the safest, with an average score of 4.3, followed by Bauer NSX and IBX (3.8). On the other hand, the Heilong was ranked as the most restrictive, with an average score of 2.3, followed by the Vix-Max (2.9). Finally, if wearing the ice hockey pad was optional, the players were most willing to wear the Bauer Vapor, with an average score of 3.7, followed by the IBX (3.6) and the Bauer NSX (3.4). Based on participant feedback, the Bauer Nsx and Bauer Vapor received the lowest restrictive ratings, with mean scores of 3.7 and 3.4, respectively. These hockey pads were also considered the most comfortable, with subjective ratings of 3.6 and 3.5, respectively. The Bauer NSX was the least restrictive among the tested hockey pads based on static and dynamic ROM measurements and was preferred in terms of comfort ratings.

The Spielman correlation method was employed to analyze the relationship between comfort and ROM for static, dynamic, and comprehensive tasks (i.e., static and dynamic tasks) measured under different pad conditions. Our study results indicated a strong correlation between the subjective comfort evaluation of ice hockey pads and upper-body ROM during static (r = 0.821, p = 0.089) and dynamic tasks (r = 0.718, p = 0.172). Furthermore, there was a highly significant (p < 0.01) and strong correlation (r = 0.975) between the comfort level of the ice hockey pads and the upper-body ROM during all tasks investigated (Figure 6). The subjective evaluation results were largely consistent with the objective data analysis. The comfort score and upper-body ROM followed the descending order: Bauer NSX > Bauer Vapor > IBX (same comfort score as Bauer Vapor) > Vix-Max > Heilong. These findings demonstrate that subjective evaluations and objective data obtained through static and dynamic tasks consistently assess ice hockey pad comfort. Therefore, ROM measurements while wearing hockey pads during static and dynamic tasks can provide objective and reliable comfort assessments. Furthermore, combining the static and dynamic testing tasks produced more accurate results for evaluating pad comfort than either a single static or dynamic task. While static ROM testing allows the assessment of the restrictive nature of pads in critical areas, dynamic ROM testing evaluates the sports performance of hockey pads during actual movement. A combination of the two tasks yielded a comprehensive and precise assessment, providing a better understanding of the strengths and weaknesses of ice hockey pads.

### Limitations of the study

This is the first study to quantify how ice hockey upper body pads affect mobility and comfort. This study had three main limitations. First, the sample size was limited to nine elite collegiate-level ice hockey players. Younger players with less strength may have more significant body motion restrictions, whereas higher level players may have fewer mobility restrictions. Consequently, an in-depth study with a larger and more diverse sample size should be conducted. Second, new ice hockey pads that were not worn or washed before the study were used. Therefore, we cannot determine how clean or “broken-in” ice hockey pads limit mobility. The fact that all Western brands of equipment sized for all S/EPs investigated did not conform to Asian body measurements may have contributed to limited mobility. The design of ice hockey pads should be improved based on a thorough understanding of the anthropometric data of Asian players. Finally, this study only assessed the effects of mobility. Future studies could introduce more physiological data, whole-body kinematic data, and real-life games to more comprehensively evaluate the effect of protective equipment on hockey player performance, including body posture and muscle fatigue, to better guide athletes in choosing the right equipment and improving their performance.

## STAR★Methods

### Key resources table

**Table:** 

REAGENT or RESOURCE	SOURCE	IDENTIFIER
**Deposited data**		

De-identified raw data of experiment	FigShare	https://doi.org/10.6084/m9.figshare.22493572

**Experimental models: Organisms/strains**		

Nine elite collegiate-level ice hockey players (eight males and one female; seven left-handed and two right-handed shooters; ages: 22.3 ± 1.3 years; height: 176.2 ± 3.9 cm; body mass: 72.5 ± 5.1 kg)	Recruited at Beijing Sport University	N/A

**Software and algorithms**		

Axis Studio 2.9	Noitom Ltd., Beijing, China	https://www.noitom.com.cn/perception-neuron-studio.html
Python 3.9	Python software foundation	http://www.python.org/
IBM SPSS Statistics for Windows, version 26.0	IBM Corporation, Armonk, N.Y., USA	https://www.ibm.com/products/spss-statistics

**Other**		

MacBook Pro 2020	Apple, Inc.	https://www.apple.com/mac/
Bauer Vapor 1X LITE	Bauer Hockey Inc.	https://www.bauer.com/
Bauer Nsx S18	Bauer Hockey Inc.	https://www.bauer.com/
Vix-Max Premium	Vik-Max Sports Equipment Inc.	http://www.vik-max.com/
Heilong Premium	Heilong International Ice & Snow Equipment Inc.	http://www.heilonggroup.com/
IBX X730	Icebreaker Sports Equipment Inc.	https://ibxhockey.com/

### Resource availability

#### Lead contact

Further information and requests for resources should be directed to and fulfilled by the lead contact, Qi Chen (chenqi@ciss.cn).

#### Materials availability

This study did not generate unique reagents or materials.

#### Data and code availability

• De-identified data have been deposited at FigShare and are publicly available as of the date of publication. Open access link is listed in the key resources table.
• This paper does not report original code.
• Any additional information required to reanalyze the data reported in this paper is available from the lead contact upon request.

### Experimental model and study participant details

#### Participants

Nine elite collegiate-level ice hockey players (Asian; eight males and one female; seven left-handed and two right-handed shooters; ages: 22.3 ± 1.3 years; height: 176.2 ± 3.9 cm; body mass: 72.5 ± 5.1 kg) from Beijing Sport University participated in this study. These players had at least 3 years of ice training, including hockey shot technique training. The exclusion criteria required participants to be able to perform experimental procedures independently and not have had any musculoskeletal or chronic neurological disorders in the past year. This study was conducted in accordance with the Declaration of Helsinki and approved by the Beijing Sport University Ethics Committee (2022194H). All the players signed an informed consent form before participating in the study.

### Method details

#### Experimental design

Five different types of ice hockey S/EPs, including the Bauer Vapor (1X LITE, constructed with carbon fiber composite material and high-density polyethylene, Bauer Hockey Inc., USA), Bauer Nsx (S18, constructed with medium and high-density polyethylene, Bauer Hockey Inc.), Vix-Max (Premium, constructed with ethylene-vinyl acetate copolymer, Vik-Max Sports Equipment Inc., China), Heilong (Premium, constructed with ethylene-vinyl acetate copolymer and polyethylene foam, Heilong International Ice & Snow Equipment Inc., China), and IBX (X730, constructed with polyurethane foam and polyethylene foam, Icebreaker Sports Equipment Inc., China) (Figure 1), were assessed in this study. S/EPs were selected considering their commercial availability, ensuring that ice hockey players could easily buy them either from a sports store or online, and that all S/EPs comply with GB/T standards. Although a quantitative assessment of the material properties of the S/EPs was not undertaken, subjective perceptions and observations indicated that Bauer Vapor, Bauer Nsx, and IBX exhibit greater lightness and flexibility than Vix-Max and Heilong, both of which utilize ethylene-vinyl acetate copolymers in their construction.

Evaluation of the ergonomic performance of PPE generally includes static and dynamic tests.^29^ The static test refers to a single degree of freedom (DOF) upper-body movement, and the dynamic test refers to authentic tasks in actual games. The test protocol of this study included nine upper-body static tasks (shoulder flexion/extension, shoulder adduction/abduction, shoulder internal/external rotation, elbow flexion, and forearm pronation/supination) and two full-body dynamic tasks (wrist and slap shots), which are the most common shooting techniques in ice hockey^31^ (Figure 2). We used a repeated measures protocol in which participants were required to perform five repetitions of static and dynamic tasks under six different pad conditions (no S/EPs as the control condition and five types of S/EPs) to examine the effect of an ice hockey pad on upper-body ROM.

The order of the six S/EP conditions was randomized (no S/EPs as the control condition and five types of S/EPs). For both tasks, measurements were obtained in the same progressive order: static to dynamic. The order of the measurements was standardized as follows: shoulder flexion, shoulder extension, shoulder adduction, shoulder abduction, shoulder internal rotation, shoulder external rotation, elbow flexion, forearm pronation, forearm supination, wrist shot, and slap shot. We provided comprehensive explanations of all tasks to every participant, ensuring their familiarity with the procedures, before the commencement of the tests. The participants were instructed to move their shoulders and elbows in the direction of the intended movement while maintaining a high level of comfort. An appropriate pad size was established in advance, following the manufacturer's sizing guidelines and factoring in each participant's height and weight. Subsequently, our biomechanical laboratory served as a venue for all the static and dynamic trials. After testing each type of S/EPs, players were asked to respond to the following statements without knowledge of the ROM measurement: (1) "This shoulder or elbow pad is comfortable"; (2) "This shoulder or elbow pad is safe"; (3) "This shoulder or elbow pad limits my shoulder or elbow motion"; and (4) "I would wear this specific shoulder or elbow pad if wearing one is optional." Responses were scored on a 1–5 Likert scale (1 = strongly disagree, 2 = disagree, 3 = unsure, 4 = agree, and 5 = strongly agree).^33^ Moreover, the assessment of the subsequent condition commenced promptly once the participant switched to new pads.

#### Instrumentation

A commercial motion capture system (Perception Neuron Studio, PNS, China) with multiple inertial measurement units (IMUs) was used to measure the upper-body kinematics during all testing trials. According to the recommendations of manufacturer,^38^ these IMUs (15.1 g; 43 × 33 × 20 mm) were placed on each body segment, i.e., the head, shoulders, upper spine, lower spine, upper arms, forearms, and hands (Figure S1). The sampling frequency was set to 96 Hz to record each trial. Several studies have demonstrated that the PNS can provide sufficiently accurate full-body kinematics for further biomechanical analysis^39^^,^^40^^,^^41^ and can detect small performance changes.^42^

### Quantification and statistical analysis

#### Data processing

Using Axis Studio 2.9, raw data obtained from the PNS were processed and converted into skeletal quaternions. A 4th-order Butterworth low-pass filter (6 Hz) was applied to the skeletal quaternion data to remove high-frequency noise. Then, we used Equation 1 to calculate the joint angle quaternions, and the YXZ rotation order^43^ was used to calculate the upper-body joint angles.

(Equation 1) $$q_{joint}={\left({q_{r}}^{b1}\right)}^{-1}⨂{q_{r}}^{b2}$$

The target joint quaternion is represented by $q_{joint}$, while the distal- and proximal-segment limb quaternions are denoted by ${q_{r}}^{b1}$ and ${q_{r}}^{b2}$, respectively. The symbol ⨂ represents quaternion multiplication.

#### Outcome measures

ROM analysis has been widely used to quantify mobility and motion patterns during the wearing of PPE.^30^ In this study, the upper-body ROM (i.e., shoulder and elbow in different motion planes) was used to describe the kinematics under six different S/EP conditions. For the static tasks, the ROM was calculated from the initial anatomical angle to the maximum angle for the given functional movement. The upper-body kinematics from both sides were then averaged, assuming that the movements of the left and right sides were balanced.^44^ For dynamic tasks, two shooting events were identified in the wrist shot (shot initiation (SI) and shot release (SR)^31^) and three in the slap shot (shot initiation (SI), swing top (ST), and shot release (SR)^32^) (Figure 2), and ROM was measured from SI to SR, SI to ST, and ST to SR, respectively. During the performance of dynamic tasks, the upper-body motion of all right-handed shooters was converted to left-handed to facilitate data comparison and analysis. In addition, the data from all trials for each participant in each condition were averaged to create a typical S/EPs trial for subsequent statistical analysis.^45^

#### Statistical analysis

The dependent variable was the upper-body ROM, and the independent variable was the ice hockey S/EPs condition (no S/EPs and five types of S/EP conditions). Descriptive statistics (M±SD) for the upper-body ROM measurements in each anatomical plane were calculated for each condition. The normality of the kinematic data distributions was tested using the Shapiro–Wilk test. Differences were analyzed using one-way repeated measures ANOVA with Greenhouse-Geisser correction,^46^ followed by a post-hoc pairwise comparison of the least significant difference for multiple comparisons^47^ if a significant main effect was found. The significance level was set at 0.05 (α = 0.05) to minimize the probability. The effect size for ANOVA was calculated using partial eta-squared ($\eta_{p}^{2}$) and considered a small effect ( 0.01 ≤ $\eta_{p}^{2}$ < 0.06), medium effect (0.06 ≤ $\eta_{p}^{2}$ < 0.14), and significant effect ($\eta_{p}^{2}$ ≥ 0.14).^48^ In addition, participants’ responses to the survey questions were analyzed using nonparametric Friedman tests, followed by post-hoc Wilcoxon signed-rank tests for paired comparisons.^49^ The Spearman correlation method was used to analyze the relationship between comfort and upper-body ROM. Correlation coefficients r within 0.1–0.29 are low correlations, 0.3–0.49 are moderate, and 0.5–1 are high correlations.^50^ The statistics were calculated using Python 3.9 and SPSS 26.0 (IBM Corporation, Armonk, NY, USA).

### Additional resources

This study was approved by the Beijing Sport University Ethics Committee (reference number: 2022194H).
