# Supplementary material for: Quantifying the effects of ice hockey upper body pads on mobility and comfort
Source: iScience. 2023 Nov 30;27(1):108606. doi: 10.1016/j.isci.2023.108606 (PMC10758976; doi:10.1016/j.isci.2023.108606)
Supplement: Document S1. Figure S1 and Tables S1–S5 [file mmc1.pdf]

iScience, Volume *27*

Supplemental information

Quantifying the effects of ice hockey upper body pads on mobility and comfort

Yiwei Wu, Yanfei Shen, Yinsheng Tian, Qi Chen, and Lixin Sun

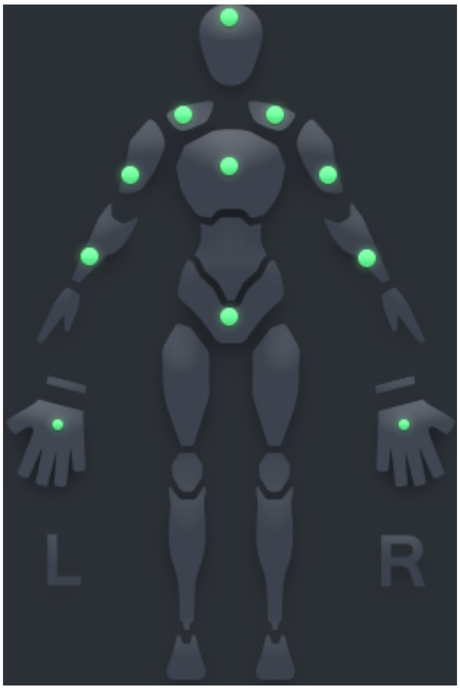

Figure S1. Placements of 11 IMUs on a participant. (Related to STAR Methods)

These IMUs were attached and strapped to each body segment of the head (1), shoulders (2), upper spine (1), lower spine (1), upper arms (2), forearms (2), and hands (2).

L, left; R, right; IMUs, inertial measurement units.

Table S1. Results of the post-hoc pairwise comparison of one-way repeated measures ANOVA between (i.e., p-value) S/EPs during all investigated static tasks. (Related to Table 1)

Variables		Shoulder						Elbow		
		**Flex**	**Ext**	**Add**	**Abd**	**Int Rot**	**Ext Rot**	**Flex**	**Pro**	**Sup**
No S/EPs	Bauer Vapor	< 0.01	0.015	0.068	0.026	0.019	0.093	< 0.01	< 0.01	< 0.01
	Bauer Nsx	< 0.01	0.124	0.194	< 0.01	< 0.01	< 0.01	< 0.01	0.016	0.169
	Vik-Max	< 0.01	< 0.01	< 0.01	< 0.01	< 0.01	< 0.01	< 0.01	< 0.01	0.026
	Heilong	< 0.01	< 0.01	0.023	< 0.01	< 0.01	< 0.01	< 0.01	< 0.01	< 0.01
	IBX	< 0.01	0.020	< 0.01	0.010	< 0.01	< 0.01	< 0.01	< 0.01	< 0.01
Bauer Vapor	Bauer Nsx	0.844	0.334	0.586	0.582	0.489	0.158	1.000	0.689	0.123
	Vik-Max	0.919	0.724	0.161	0.019	< 0.01	0.023	0.288	0.752	0.513
	Heilong	0.377	0.230	0.624	0.010	< 0.01	0.179	1.000	0.948	0.799
	IBX	0.583	0.898	0.170	0.672	0.033	0.096	1.000	0.329	0.912
Bauer Nsx	Vik-Max	0.767	0.190	0.054	0.067	0.012	0.364	1.000	0.475	0.366
	Heilong	0.282	0.034	0.303	0.038	0.029	0.943	1.000	0.641	0.075
	IBX	0.724	0.401	0.058	0.897	0.139	0.791	1.000	0.172	< 0.01
Vik-Max	Heilong	0.433	0.393	0.356	0.796	0.723	0.328	1.000	0.802	0.365
	IBX	0.516	0.631	0.975	0.051	0.271	0.519	0.519	0.507	0.445
Heilong	IBX	0.156	0.185	0.372	0.029	0.452	0.737	1.000	0.362	0.885

S/EPs; shoulder and elbow pads; ROM, range of motion; ANOVA, analysis of variance; Flex, flexion; Ext, extension; Add, adduction; Abd, abduction; Int Rot, internal rotation; Ext Rot, external rotation; Pro, pronation; Sup, supination.

Table S2. Results of the post-hoc pairwise comparison of one-way repeated measures ANOVA between (i.e., p-value) S/EPs during the wrist shot. (Related to Related to Table 2)

Variables		Shoulder						Elbow			
		**Flex/Ext L**	**Add/Abd L**	**Int/Ext Rot L**	**Flex/Ext R**	**Add/Abd R**	**Int/Ext Rot R**	**Flex L**	**Pro/Sup L**	**Flex R**	**Pro/Sup R**
No S/EPs	Bauer Vapor	< 0.01	0.641	0.411	0.015	0.714	0.088	< 0.01	0.232	0.475	0.630
	Bauer Nsx	< 0.01	0.488	0.333	< 0.01	0.063	0.213	< 0.01	0.789	0.251	0.699
	Vik-Max	0.018	0.183	0.196	< 0.01	0.949	0.016	< 0.01	0.376	0.145	0.664
	Heilong	0.433	0.495	0.926	0.046	0.350	0.936	< 0.01	0.061	0.708	0.863
	IBX	0.215	0.396	0.094	0.017	0.904	0.766	0.178	0.118	0.725	0.574
Bauer Vapor	Bauer Nsx	0.656	0.819	0.882	0.619	0.129	0.634	0.479	0.350	0.660	0.925
	Vik-Max	0.649	0.381	0.630	0.758	0.666	0.446	0.696	0.750	0.449	0.362
	Heilong	0.040	0.828	0.465	0.628	0.567	0.075	0.626	0.478	0.279	0.757
	IBX	0.105	0.700	0.382	0.949	0.805	0.047	0.039	0.704	0.289	0.936
Bauer Nsx	Vik-Max	0.369	0.516	0.739	0.850	0.055	0.218	0.750	0.536	0.749	0.412
	Heilong	0.014	0.991	0.380	0.329	0.335	0.186	0.235	0.104	0.131	0.829
	IBX	0.041	0.875	0.467	0.576	0.080	0.125	0.162	0.192	0.137	0.861
Vik-Max	Heilong	0.104	0.508	0.229	0.430	0.319	0.013	0.382	0.306	0.070	0.545
	IBX	0.238	0.621	0.692	0.710	0.854	< 0.01	0.089	0.486	0.073	0.322
Heilong	IBX	0.643	0.866	0.113	0.674	0.414	0.828	0.012	0.741	0.982	0.696

S/EPs; shoulder and elbow pads; ANOVA, analysis of variance; Flex/Ext L, flexion/extension left; Add/Abd L, adduction/abduction left; Int/Ext Rot L, internal/external rotation left; Flex/Ext R, flexion/extension right; Add/Abd R, adduction/abduction right; Int/Ext Rot R, internal/external rotation right; Flex L, flexion left; Pro/Sup L, pronation/supination left; Flex R, flexion right; Pro/Sup R, pronation/supination right.

Table S3. Results of the post-hoc pairwise comparison of one-way repeated measures ANOVA between (i.e., p-value) S/EPs during the SI to ST of a slap shot. (Related to Table 3)

**Variables**		**Shoulder**						**Elbow**			
		**Flex/Ext L**	**Add/Abd L**	**Int/Ext Rot L**	**Flex/Ext R**	**Add/Abd R**	**Int/Ext Rot R**	**Flex L**	**Pro/Sup L**	**Flex R**	**Pro/Sup R**
No S/EPs	Bauer Vapor	0.348	0.778	0.938	0.522	0.492	0.854	0.212	0.037	0.145	0.536
	Bauer Nsx	0.087	0.529	0.188	0.189	0.880	0.282	< 0.01	0.014	0.813	0.949
	Vik-Max	0.332	0.025	0.806	0.164	0.437	< 0.01	< 0.01	0.134	0.902	0.773
	Heilong	0.097	0.807	0.156	< 0.01	0.145	< 0.01	< 0.01	< 0.01	0.609	0.984
	IBX	0.936	0.077	0.609	0.855	0.093	0.073	< 0.01	0.227	0.906	0.765
Bauer Vapor	Bauer Nsx	0.427	0.728	0.164	0.494	0.403	0.210	0.139	0.685	0.220	0.578
	Vik-Max	0.975	0.047	0.746	0.446	0.928	< 0.01	0.074	0.535	0.116	0.366
	Heilong	0.458	0.969	0.135	0.017	0.433	< 0.01	0.064	0.160	0.339	0.549
	IBX	0.390	0.134	0.556	0.646	0.309	0.049	< 0.01	0.359	0.179	0.748
Bauer Nsx	Vik-Max	0.445	0.097	0.281	0.936	0.355	0.109	0.744	0.307	0.719	0.726
	Heilong	0.958	0.699	0.916	0.077	0.109	0.114	0.695	0.313	0.783	0.965
	IBX	0.102	0.246	0.414	0.256	0.068	0.458	0.141	0.189	0.905	0.814
Vik-Max	Heilong	0.476	0.043	0.238	0.091	0.487	0.984	0.947	0.046	0.526	0.758
	IBX	0.373	0.604	0.790	0.2248	0.354	0.379	0.247	0.764	0.810	0.558
Heilong	IBX	0.113	0.125	0.357	< 0.015	0.814	0.390	0.275	0.023	0.693	0.780

S/EPs; shoulder and elbow pads; ANOVA, analysis of variance; SI, shot initiation; ST, swing top; Flex/Ext L, flexion/extension left; Add/Abd L, adduction/abduction left; Int/Ext Rot L, internal/external rotation left; Flex/Ext R, flexion/extension right; Add/Abd R, adduction/abduction right; Int/Ext Rot R, internal/external rotation right; Flex L, flexion left; Pro/Sup L, pronation/supination left; Flex R, flexion right; Pro/Sup R, pronation/supination right.

Table S4. Results of the post-hoc pairwise comparison of one-way repeated measures ANOVA between (i.e., p-value) S/EPs during the ST to SR of a slap shot. (Related to Table 3)

Variables		Shoulder						Elbow			
		**Flex/Ext L**	**Add/Abd L**	**Int/Ext Rot L**	**Flex/Ext R**	**Add/Abd R**	**Int/Ext Rot R**	**Flex L**	**Pro/Sup L**	**Flex R**	**Pro/Sup R**
No S/EPs	Bauer Vapor	< 0.01	0.911	0.656	0.916	0.015	0.453	0.017	0.424	0.089	0.114
	Bauer Nsx	0.204	0.312	0.105	0.568	0.0307	0.641	0.192	0.196	0.978	0.922
	Vik-Max	0.210	0.987	0.057	0.987	0.021	< 0.01	< 0.01	0.762	0.682	0.509
	Heilong	0.140	0.576	< 0.01	0.118	< 0.01	0.242	< 0.01	0.170	0.897	0.923
	IBX	0.432	0.436	0.160	0.308	0.021	< 0.01	< 0.01	0.938	0.381	0.404
Bauer Vapor	Bauer Nsx	0.069	0.367	0.234	0.499	0.773	0.775	0.253	0.612	0.094	0.137
	Vik-Max	0.067	0.899	0.138	0.904	0.899	0.037	0.792	0.273	0.191	0.348
	Heilong	0.106	0.654	0.012	0.096	0.416	0.669	0.213	0.557	0.115	0.137
	IBX	0.023	0.504	0.331	0.2614	0.906	0.038	0.651	0.381	0.397	0.445
Bauer Nsx	Vik-Max	0.985	0.304	0.762	0.579	0.871	0.019	0.162	0.114	0.702	0.575
	Heilong	0.831	0.647	0.159	0.311	0.273	0.476	0.020	0.935	0.918	0.999
	IBX	0.621	0.813	0.822	0.649	0.864	0.019	0.114	0.171	0.396	0.460
Vik-Max	Heilong	0.816	0.565	0.265	0.121	0.349	0.090	0.323	0.097	0.779	0.573
	IBX	0.635	0.427	0.598	0.315	0.993	0.991	0.851	0.822	0.638	0.859
Heilong	IBX	0.480	0.824	0.104	0.573	0.353	0.092	0.422	0.148	0.454	0.460

S/EPs; shoulder and elbow pads; ANOVA, analysis of variance; SR, shot release; ST, swing top; Flex/Ext L, flexion/extension left; Add/Abd L, adduction/abduction left; Int/Ext Rot L, internal/external rotation left; Flex/Ext R, flexion/extension right; Add/Abd R, adduction/abduction right; Int/Ext Rot R, internal/external rotation right; Flex L, flexion left; Pro/Sup L, pronation/supination left; Flex R, flexion right; Pro/Sup R, pronation/supination right.

Table S5. Subjective evaluation scores of different types of S/EPs during both static and dynamic tasks. (Related to Figure 5)

Variables	Bauer Vapor	Bauer Nsx	Vik-Max	Heilong	IBX
This shoulder or elbow pad is comfortable	3.5 ± 0.7	3.6 ± 1.0	2.9 ± 0.8	2.3 ± 0.8	3.5 ± 1.0
This shoulder or elbow pad is safe	4.3 ± 0.6	3.8 ± 1.1	2.8 ± 1.1	2.9 ± 1.2	3.8 ± 0.9
This shoulder or elbow pad limits my shoulder or elbow motion	3.4 ± 0.9	3.7 ± 0.9	2.9 ± 0.7	2.3 ± 0.7	3.2 ± 0.7
I would wear this specific shoulder or elbow pad if wearing one is optional	3.7 ± 1.2	3.4 ± 1.2	2.3 ± 0.7	2.0 ± 0.9	3.6 ± 0.9

S/EPs; shoulder and elbow pads
